# A Gene Panel, Including *LRP12*, Is Frequently Hypermethylated in Major Types of B-Cell Lymphoma

**DOI:** 10.1371/journal.pone.0104249

**Published:** 2014-09-16

**Authors:** Nicole Bethge, Hilde Honne, Kim Andresen, Vera Hilden, Gunhild Trøen, Knut Liestøl, Harald Holte, Jan Delabie, Guro E. Lind, Erlend B. Smeland

**Affiliations:** 1 Department of Immunology, Institute for Cancer Research, Oslo University Hospital, Oslo, Norway; 2 Centre for Cancer Biomedicine, University of Oslo, Oslo, Norway; 3 Department of Cancer Prevention, Institute for Cancer Research, Oslo University Hospital, Oslo, Norway; 4 Department of Pathology, Oslo University Hospital, Oslo, Norway; 5 Department of Informatics, University of Oslo, Oslo, Norway; 6 Department of Oncology, The Norwegian Radium Hospital, Oslo University Hospital, Oslo, Norway; The University of North Carolina at Chapel Hill, United States of America

## Abstract

Epigenetic modifications and DNA methylation in particular, have been recognized as important mechanisms to alter gene expression in malignant cells. Here, we identified candidate genes which were upregulated after an epigenetic treatment of B-cell lymphoma cell lines (Burkitt's lymphoma, BL; Follicular lymphoma, FL; Diffuse large B-cell lymphoma, DLBCL activated B-cell like, ABC; and germinal center like, GCB) and simultaneously expressed at low levels in samples from lymphoma patients. Qualitative methylation analysis of 24 candidate genes in cell lines revealed five methylated genes (*BMP7, BMPER*, *CDH1*, *DUSP4* and *LRP12*), which were further subjected to quantitative methylation analysis in clinical samples from 59 lymphoma patients (BL, FL, DLBCL ABC and GCB; and primary mediastinal B-cell lymphoma, PMBL). The genes *LRP12* and *CDH1* showed the highest methylation frequencies (94% and 92%, respectively). *BMPER* (58%), *DUSP4* (32%) and *BMP7* (22%), were also frequently methylated in patient samples. Importantly, all gene promoters were unmethylated in various control samples (CD19+ peripheral blood B cells, peripheral blood mononuclear cells and tonsils) as well as in follicular hyperplasia samples, underscoring a high specificity. The combination of *LRP12* and *CDH1* methylation could successfully discriminate between the vast majority of the lymphoma and control samples, emphasized by receiver operating characteristic analysis with a c-statistic of 0.999. These two genes represent promising epigenetic markers which may be suitable for monitoring of B-cell lymphoma.

## Introduction

B-cell lymphoma comprises diverse neoplasms that are typed according to different B-cell developmental stages [1]. Chromosomal translocations involving the immunoglobulin (Ig) gene loci and oncogenes, such as *MYC*, *BCL1* and *BCL2*, are typical for B-cell lymphoma. In addition, a broad pattern of other acquired genetic changes have been described, although considerable heterogeneity exists even within each lymphoma type [2]. During the past decades it has become evident that alterations in the methylome can be found in nearly all cancer types [3], [4] and this is now considered to be important in the pathogenesis of most cancer types [5]. DNA promoter hypermethylation has been described in a variety of genes and represents one important mechanism for the loss of tumor suppressor gene activity [6], [7]. Inactivation of tumor suppressor genes by methylation has also been shown in lymphoma, e.g. the cyclin-dependent kinase inhibitors *CDKN2A* and *CDKN2B* [8], the TP53 homologue *TP73* and the death-associated protein kinase *DAPK1* [9]. Recently, several large-scale studies have been used to discover additional aberrantly methylated genes in lymphoma, although at various frequencies [10], [11]. Interestingly, some of the these hypermethylated genes have been shown to predict the outcome of therapy [12], and it was recently shown that DLBCL with hypermethylation of the *MGMT* promoter had a favorable outcome [13].

The aim of this study was to identify novel methylated genes and to analyze their promoter methylation status in major types of B-cell lymphomas (diffuse large B cell-, follicular- and Burkitt's lymphoma).

## Methods

### Primary samples

DNA from 59 patients diagnosed with B-cell lymphoma (germinal center B cell–like (GCB) (n = 16) and activated B cell–like (ABC) (n = 18) subtypes of diffuse large B cell lymphoma (DLBCL), primary mediastinal B-cell lymphoma (PMBL) (n = 6), follicular lymphoma (FL) (n = 12) and Burkitt's lymphoma (BL) (n = 7)) were included in the study. In addition, DNA from several non-malignant sources, which are referred to as control samples (n = 49) were included as well; i.e. normal B cells isolated from buffy coat with CD19^+^ Dynabeads (Invitrogen), *n* = 20; peripheral blood mononuclear cells, *n* = 10; and tonsils, *n* = 10; in addition to follicular hyperplasia samples; *n* = 9. The 59 patients included in this study were observed for on average 36 months (median value) after diagnosis. During this time, eight out of the 59 patients (14%) died.

### Ethical statement

The study was approved by the Regional Committees for Medical and Health Research Ethics, Region Eastern Norway (S-05145) and was performed in accordance with the Declaration of Helsinki. Informed consent was signed by all patients included in this study.

### Epigenetic treatment of lymphoma cell lines

Twelve B-cell lymphoma cell lines (BL: BL41, Raji and Ramos (Deutsche Sammlung von Microorganismen und Zellkulturen Gmbh; DSMZ); DLBCL ABC: HLY-1 (T.Saati), OciLy3, and OciLy10 (L. Staudt); DLBCL GCB: OciLy7 and SUDHL4 (L. Staudt), SUDHL6 (DSMZ); and FL: K422, SC-1 and ROS50 (DSMZ) were cultured with and without a combination of epigenetic drugs; the demethylating reagent 5-aza-2′deoxycytidine (aza; 1 µM for 72 h) and the histone deacetylase inhibitor trichostatin A (TSA; 0.5 µM added the last 12 h). Culturing conditions are described in File S1.

### Gene expression profiling

A stepwise approach was applied to identify potentially methylated target genes. Epigenetic drug treated cell lines and their untreated counterparts were analyzed with the Applied Biosystems Human Genome Survey Microarray following manufacturer's protocol. Post-processing and normalization was performed with the R-script “ABarray” and Bioconductor (GSE46064). Furthermore, gene expression data from 480 B-cell lymphomas were available from the Leukemia Lymphoma Molecular Profiling Project (LLMPP) (BL *n* = 24, GSE 4732 [14]; DLBCL ABC *n* = 168, GSE10846 [15]; DLBCL GC *n* = 97, GSE10846 [15]; FL *n* = 191, unpublished). The gene expression profile of each lymphoma type was compared to the average expression of the remaining B-cell lymphoma types. We selected candidate genes by the following criteria; gene expression of B-cell lymphoma patients had to be at least 2-fold downregulated in one B-cell lymphoma type compared to the other types. At the same time, the genes had to be at least 0.5-fold upregulated after epigenetic drug treatment of cell lines representing the same type of lymphoma. Furthermore, the average response of epigenetic drug treatment of cell lines of the given lymphoma type should be 0.5-fold higher compared to the average response of the remaining cell lines.

### Analysis of promoter methylation status in cell lines

Genes harboring a CpG-Island in their promoter region (CpG-Island Searcher Software, [16]), were analyzed by methylation specific PCR (MSP) in B-cell lymphoma cell lines and CD19^+^ B-cells. MSP was performed as previously described [17] and each reaction was run twice for an independent validation. All primers amplifying methylated or unmethylated loci were designed using the Methyl Primer Express 1.0 (Applied Biosystems; sequences listed in Table S1 in File S1). DNA from B-cell lymphoma cell lines and CD19^+^ B-cells was isolated with the AllPrep DNA/RNA/protein Kit from Qiagen. For each sample, 1.3 µg DNA was bisulfite treated with the EpiTect bisulfite kit (Qiagen).

### Direct bisulfite sequencing

Primers for bisulfite sequencing were designed using Methyl Primer Express 1.0 (Applied Biosystems) to amplify the respective MSP primer binding sites for each gene of interest (Primer sequences are provided in Table S1 in File S1). Bisulfite sequencing was performed as previously described [17]. By comparing the peak height of the cytosine signal with the sum of the cytosine and thymine peak height signals, the approximate amount of methyl cytosine of each CpG site was calculated. CpG sites with ratio between: 0 and 0.20 were scored as unmethylated, 0.21 to 0.80 were scored as partially methylated, whereas 0.81 to 1.0 was considered to be fully methylated.

### Quantitative methylation-specific polymerase chain reaction (qMSP)

Primers and probes for qMSP, which bind bisulfite treated and methylated DNA, were designed with the Primer Express 3.0 Software (Applied Biosystems; assays listed in Table S1 in File S1). All qMSP reactions were performed in triplicates using a 7900HT Fast Real-Time PCR System (Applied Biosystems) and analyzed with the sequence detector system 2.3 (Applied Biosystems), as previously described [17]. Briefly, normalization for DNA input was performed using the ALU-C4 as a reference gene [18], and the quantity of methylated DNA in each sample was determined using a standard curve of bisulfite treated universal methylated DNA (Chemicon, Millipore).

### Statistics

Statistical analyses were carried out using GraphPad Prism 6 (GraphPad Software, Inc, San Diego, CA, USA). A Mann- Whitney test was used to compare the PMR values of the candidate genes between the various sample groups testes. A Fisher's exact test was used for analyzing potential differences in the number of methylated samples between the test and validation series. All *P*-values were derived from two-sided tests, and *P*≤0.05 was considered to be statistically significant. Finally, Receiver operating characteristics (ROC) curve analyses were used to evaluate the performance of the methylation biomarkers.

## Results

### Identification of genes upregulated after epigenetic treatment of B-cell lymphoma cell lines and expressed at a low level in lymphoma patients

We analyzed the top 24 candidate genes, which were upregulated after epigenetic treatment of cell lines and simultaneously expressed at low levels in lymphoma samples of the corresponding type. These candidates were analyzed by MSP in 12 B-cell lymphoma cell lines and CD19+ peripheral blood B cells from healthy donors. The gene promoters of *BMPER, CDH1* and *LRP12* were methylated in all analyzed B-cell lymphoma cell lines across all subtypes (Table 1). In addition, the following genes had a high promoter methylation frequency *DUSP4* (92%); *CCL22*, *CLU and NPY1R* (83%); *BCL2L10, PTPRG* and *UCHL1* (75%). Of note, *BMP7* was methylated in all three DLBCL ABC cell lines and in two of three FL cell lines, but not in cell lines derived from BL or DLBCL GCB (Table 1). It was the only gene showing a subtype-specific methylation pattern in DLBCL cell lines.

**Table 1:** Methylation status of candidate genes in 12 B-cell lymphoma cell lines.

cell line/gene	BL (n = 3; %)	DLBCL ABC (n = 3; %)	DLBCL GCB (n = 3; %)	FL (n = 3; %)	all types combined
**BMPER**	100%	100%	100%	100%	100%
**CDH1**	100%	100%	100%	100%	100%
**LRP12**	100%	100%	100%	100%	100%
**DUSP4**	100%	100%	100%	66%	92%
**CCL22**	100%	66%	66%	100%	83%
**CLU**	100%	66%	66%	100%	83%
**NPY1R**	100%	100%	66%	66%	83%
**BCL2L10**	100%	66%	66%	66%	75%
**PTPRG**	100%	33%	66%	100%	75%
**UCHL1**	66%	100%	66%	66%	75%
**SGPP2**	66%	33%	66%	100%	67%
**BMP7**	0%	100%	0%	66%	42%
**HBEGF**	66%	33%	33%	0%	33%
**BCL2**	33%	0%	0%	0%	8%
**PRKAR2B**	0%	0%	33%	0%	8%
**CALR**	0%	0%	0%	0%	0%
**GPSM2**	0%	0%	0%	0%	0%
**ICOSLG**	0%	0%	0%	0%	0%
**KLF13**	0%	0%	0%	0%	0%
**MAP3K3**	0%	0%	0%	0%	0%
**MAPK81P3**	0%	0%	0%	0%	0%
**MYBL1**	0%	0%	0%	0%	0%
**SNX22**	0%	0%	0%	0%	0%
**XRCC4**	0%	0%	0%	0%	0%

Only candidate genes which have a CpG island in their promoter region have been analyzed by MSP. Candidate genes for each lymphoma type have been analyzed in 12 B-cell lymphoma cell lines (three cell lines per type). Genes have been sorted by the combined methylation frequency (brackets) across all lymphoma cell lines. A cell line has been considered as methylated when it was partially or fully methylated. Abbreviations: BL, Burkitt's lymphoma; DLBCL ABC, activated B-cell like diffuse large B-cell lymphoma; DLBCL GCB, germinal centre B-cell like diffuse large B-cell lymphoma; and FL, follicular lymphoma.

### Bisulfite sequencing of the *BMP7*-, *BMPER*-, *CDH1*-, *DUSP4*- and *LRP12*-promoter

**Figure 1:**
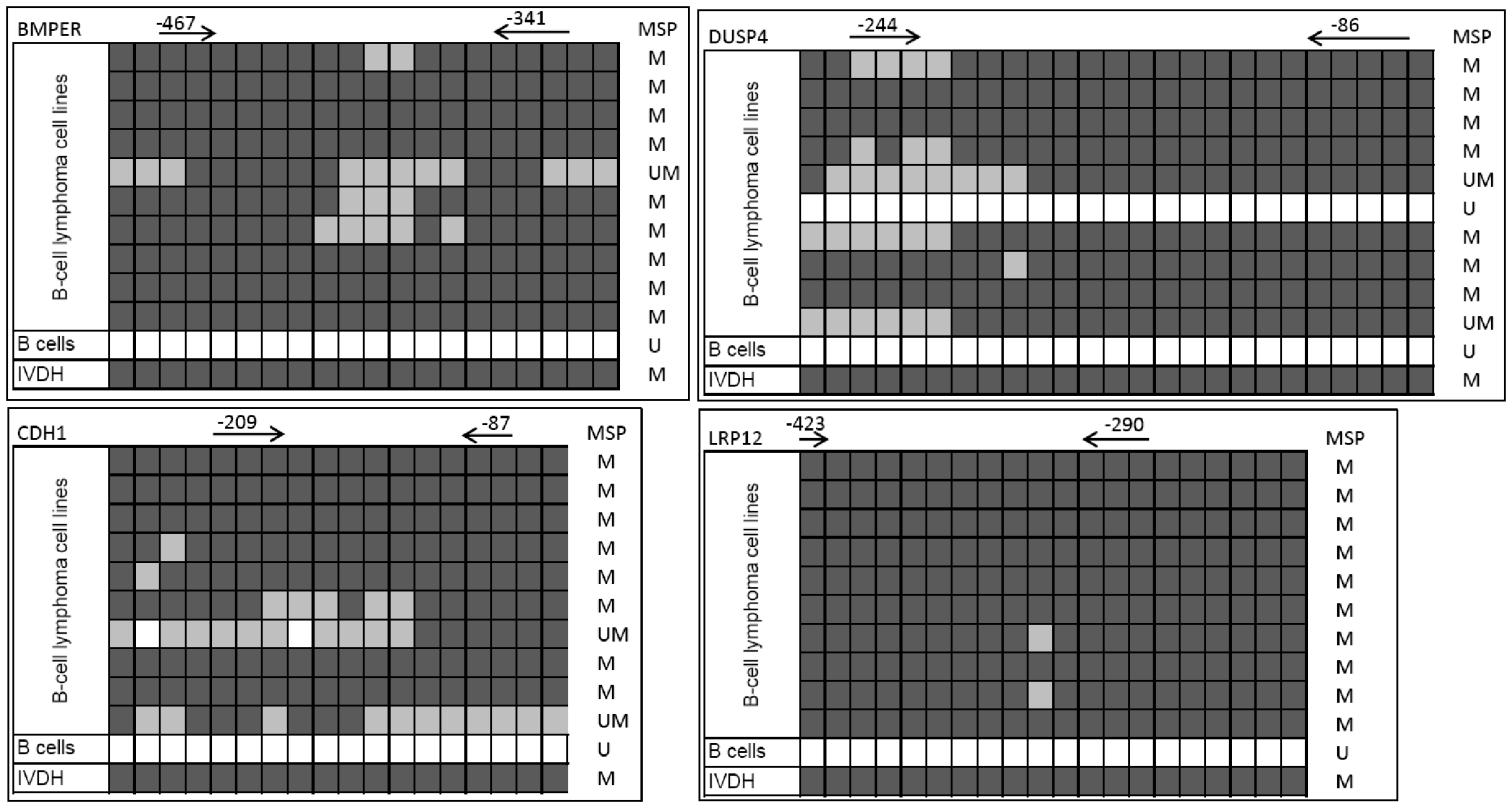
Bisulfite sequencing of individual CpG sites in the promoter regions of *BMPER, CDH1, DUSP4* and *LRP12*. CpG sites are represented by a box, a methylated site is symbolized by a dark grey box, a partially methylated CpG site is colored in gray and a white box represents an unmethylated CpG site. MSP-primer binding sites are indicated with an arrow above the CpG site. The distance from transcription start to the first base of the primer is indicated by the number above the arrow.

Before designing qMSP primers and probes, we used direct bisulfite sequencing to analyze the methylation status of individual CpG sites in the promoter regions of the five most promising candidates (*BMP7*, *BMPER*, *CDH1*, *DUSP4* and *LRP12*). In general, the bisulfite sequencing confirmed complete bisulfite conversion since all non-methylated cytosines were converted to thymine. All cell lines, which showed partial or complete methylation as assessed by MSP, revealed partially or fully methylated CpG-sites in the region for MSP-primer binding. Furthermore, B cells from healthy donors were confirmed to be negative for promoter CpG-island methylation by bisulfite sequencing (Figure 1, *BMP7* can be found in Figure S1 in File S1).

### DNA methylation analysis of lymphoma and healthy donor samples

**Figure 2:**
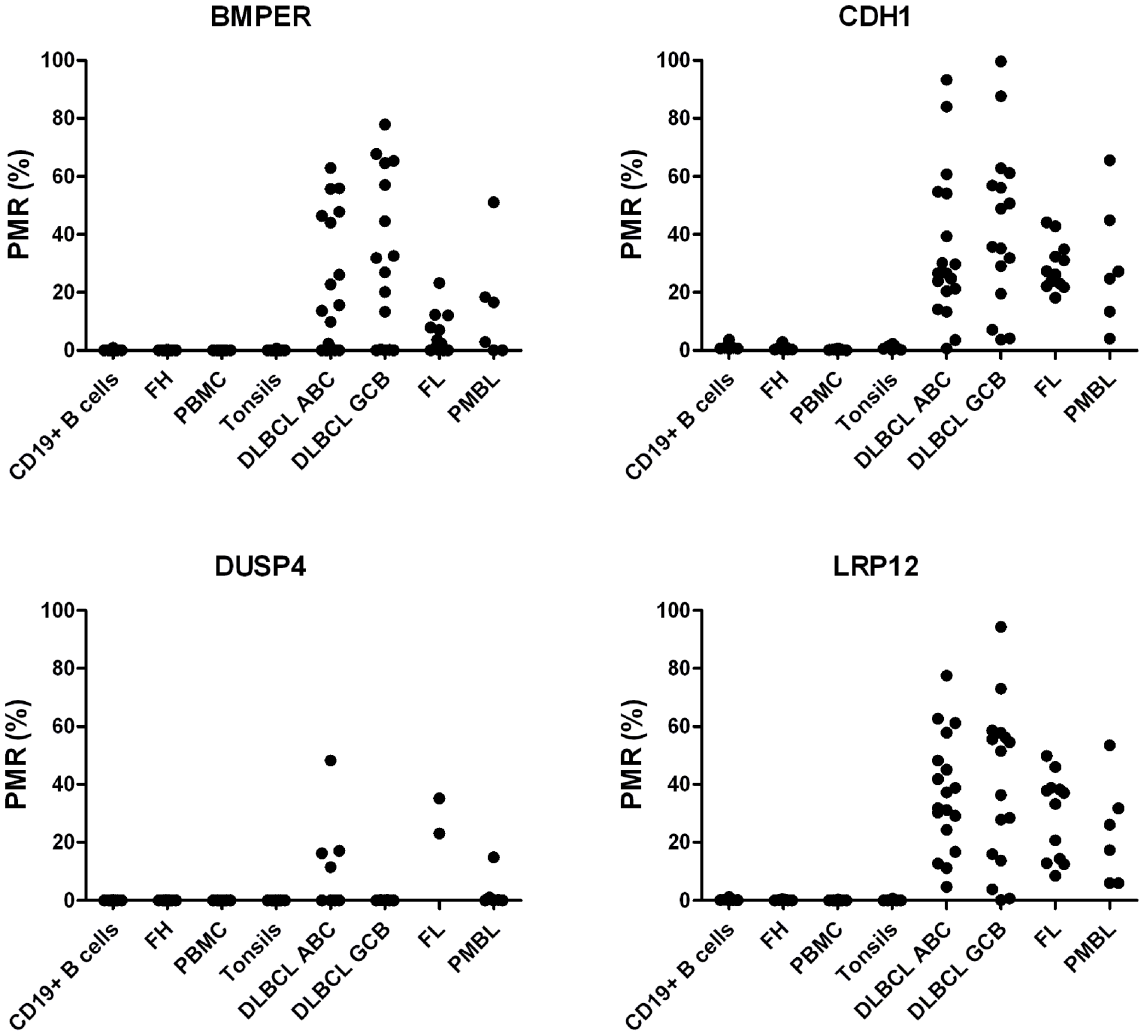
Methylation status of *BMPER, CDH1, DUSP4* and *LRP12* assessed by qMSP. Percent promoter methylation in control samples (CD19+ B cells, PMBC, tonsils); follicular hyperplasia samples (FH) and lymphoma patient samples. Each dot represents one sample. Abbreviations: BL, Burkitt's lymphoma; DLBCL ABC, activated B-cell like diffuse large B-cell lymphoma; DLBCL GCB, germinal centre B-cell like diffuse large B-cell lymphoma; FL, follicular lymphoma; and PMBL, primary mediastinal B-cell lymphoma; PMR, percent methylated reference.

We next analyzed *BMPER*, *CDH1*, *DUSP4* and *LRP12* promoter methylation in clinical samples by qMSP in a test and validation series. The lymphoma patients included in the test series showed methylation frequencies of 93%, 90% and 60% for *CDH1*, *LRP12* and *BMPER*, respectively. Due to a limited access to patient material, *DUSP4* was only analyzed in the validation series. The analyzed control samples showed low PMR values, ranging from 0–3.7%. We used the highest PMR value obtained from the analyzed control samples to set a threshold (4%) for scoring methylation positive samples. For the test and validation series, no statistically significant differences were seen for neither the number of methylated tumors nor for the level of PMR values (Table S2 in file S1). The promoter methylation of *LRP12*, *CDH1, BMPER* and *DUSP4* was 100%, 91%, 55%, and 32% across all analyzed lymphoma types included in the validation series, respectively (Table 2 and Figure 2). For *BMPER*, *CDH1* and *LRP12* statistically significant differences in the PMR values were seen between several of the lymphoma groups analyzed, as well as in comparison with the control samples (Table S2 in file S1).

**Table 2:** Methylation frequency of the analyzed lymphoma samples.

Patients/genes		BL	DLBCL ABC	DLBCL GCB	FL	PMBL	All types combined
Test series	**BMP7**	0/7 (0%)	3/10 (30%)	3/10 (30%)	2/10 (20%)	n.a.	8/37 (22%)
	**BMPER**	n.a.	6/10 (60%)	7/10 (70%)	5/10 (50%)	n.a.	18/30 (60%)
	**CDH1**	n.a.	9/10 (90%)	9/10 (90%)	10/10 (100%)	n.a.	28/30 (93%)
	**DUSP4**	n.a.	n.a.	n.a.	n.a.	n.a.	n.a.
	**LRP12**	n.a.	10/10 (100%)	7/10 (70%)	10/10 (100%)	n.a.	27/30 (90%)
Validation series	**BMP7**	n.a.	n.a.	n.a.	n.a.	n.a.	n.a.
	**BMPER**	n.a.	5/8 (63%)	4/6 (67%)	0/2 (0%)	3/6 (50%)	12/22 (55%)
	**CDH1**	n.a.	7/8 (88%)	6/6 (100%)	2/2 (100%)	5/6 (84%)	20/22 (91%)
	**DUSP4**	n.a.	4/8 (50%)	0/6 (0%)	2/2 (100%)	1/6 (17%)	7/22 (32%)
	**LRP12**	n.a.	8/8 (100%)	6/6 (100%)	2/2 (100%)	6/6 (100%)	22/22 (100%)

Gene promoters have been analyzed by qMSP in five different lymphoma types. The methylation frequency is given in brackets for each lymphoma type and as a combination of all lymphoma types (last column). Abbreviations: BL, Burkitt's lymphoma; DLBCL ABC, activated B-cell like diffuse large B-cell lymphoma; DLBCL GCB, germinal centre B-cell like diffuse large B-cell lymphoma; FL, follicular lymphoma; n.a., not analyzed; and PMBL, primary mediastinal B-cell lymphoma.

The *BMP7*-promoter methylation status was analyzed by qMSP in 37 lymphoma samples and CD19^+^ B-cells from 10 healthy donors. The promoter methylation of *BMP7* was 0%, 20%, 30% and 30% in BL, FL, DLBCL ABC and DLBCL GCB, respectively. Thus, the subtype specific methylation pattern seen in DLBCL cell lines could not be confirmed in patient samples. The promoter of *BMP7* showed no methylation in control samples (Figure S2 in file S1). Interestingly, *DUSP4* was methylated in 50% of DLBCL ABC and showed no methylation in DLBCL GCB.

### Receiver Operating Characteristics (ROC) curves

**Figure 3:**
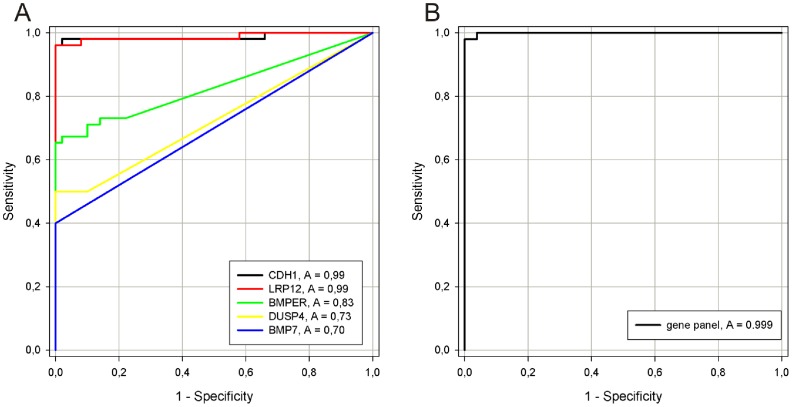
Receiver Operating Characteristics (ROC) curves for individual and combined markers in lymphoma patients versus healthy donors. The area under the ROC curve (AUC) represents how accurate the individual and combined biomarkers can discriminate between lymphoma and control samples. A) Lymphoma patients versus controls for individual genes. B) Lymphoma patients versus controls for the combined gene panel.

We used the PMR values obtained for lymphomas and healthy controls from the qMSP analysis as input in the receiver operating characteristics (ROC) curves. *BMP7*, *BMPER*, *CDH1*, *DUSP4* and *LRP12* showed an individual area under the curve (AUC) of 0.70, 0.83, 0.99, 0.74 and 0.99 (Figure 3a). By combining the panel (by summarizing the PMR values) we could discriminate all lymphoma samples (BL, DLBCL ABC, DLBCL GCB, FL and PMBL), except one, from the various control samples (B cells, PBMC and tonsils) and follicular hyperplasia as shown by an AUC of 0.999 (Figure 3b).

## Discussion

In the present study we identified genes frequently methylated in B-cell lymphoma by a stepwise approach. Genes, which were upregulated after epigenetic drug treatment of a panel of B-cell lymphoma cell lines and expressed at a low level in lymphoma patient samples, were subjected to promoter methylation analyses in cell lines. Frequently methylated genes were further analyzed in clinical samples. We focused on major types of B-cell lymphoma (BL, DLBCL ABC, DLBCL GCB, FL and PMBL) and demonstrated that the gene promoters of *LRP12 (94%), CDH1 (92%)*, and *BMPER* (58%) were methylated at a high frequency, whereas *DUSP4* and *BMP7* showed lower methylation frequencies (32% and 22%, respectively). Combined, these genes could successfully discriminate the vast majority (98%) of lymphoma samples from controls and follicular hyperplasia samples, as shown by receiver operating characteristics with an AUC of 0.999.

We found *LPR12* and *CDH1* to be the most frequently methylated genes in our study. Of note, *CDH1* has previously been shown to be frequently methylated in various cancer types [19], [20], including lymphoma [21], [22] and leukemia [23], [24] and functions as a tumor suppressor by inhibiting proliferation and invasion [25]. However, to the best of our knowledge, this is the first time *BMPER*, *BMP7*, *DUSP4* and *LRP12* are reported to be methylated in lymphoma. From previous studies, *DUSP4* and *BMP7* are known to be methylated in gliomas [26] and gastric- and prostate cancer [27], [28], respectively. Even though large scale methylation studies have been performed on different lymphoma types increasing the knowledge about methylated genes, we were able to discover novel methylated genes using a cell line based approach and studying FL, DLBCL and BL at the same time. This is in contrast to other studies focusing mainly on one lymphoma type [10], [11].

Alterations of the TGF-β/BMP signaling pathways are frequently found in human cancer [29]. In addition, TGF-β often acts as a tumor suppressor during early stages of carcinogenesis; however, at later stages it may act as a tumor promoter [30]. Interestingly, three of the methylated genes identified in the present study are related to these signaling pathways. The *LRP12 gene*, that had the highest methylation frequency, encodes a low density lipoprotein receptor-related protein. A role in signal transduction is proposed, since the cytoplasmic c-terminus of this protein can interact among others with SARA (Smad anchor receptor activation), which is reported to interact with the TGF-β and BMP pathways [31]. Moreover, the gene *BMPER*, which encodes for BMP endothelial cell precursor-derived regulator, was frequently methylated in our lymphoma samples. BMPER is a secreted factor, which modulates BMP activity, showing both pro- and anti-BMP effects [32], [33]. In addition, *BMP7*, which is methylated in 23% of the analyzed patients, as well as *BMP6*, which previously has been reported to be frequently methylated in lymphoma [12], are members of the TGF-β superfamily of cytokines. In concordance with other cancer types the TGF-β-receptor II has also been reported to be methylated in B-cell lymphoma cell lines [34]–[36]. Taken together, the data presented here indicates that alterations within the TGF-β and/or BMP signaling in lymphoma could be due to promoter hypermethylation-induced downregulation of essential TGF-β/BMP signaling pathway components.

The usage of a DNA methylation based biomarker in either primary biopsies or various body fluids has already been shown for several cancer types [37]. Of note, detection of *DLC1* methylation in primary lymphoma biopsies and plasma samples from the same patients, showed a concordance of 80% [38], indicating a possible use of plasma analyses as well. These data are encouraging, but further studies are needed to establish the degree of methylation in cell free circulating DNA of lymphoma patients. The *LRP12* and *CDH1* genes have a great potential as DNA methylation biomarkers for lymphoma, since these were able to discriminate with a high sensitivity and specificity lymphoma patients from the various control samples and follicular hyperplasia. In addition, DNA methylation is an early event in tumor development; a DNA methylation based biomarker could be used to monitor lymphoma patients for relapse. Furthermore, biomarkers, which can predict the response to therapy are of great value and has been shown for various methylated genes [39]–[42]. Of note, epigenetic modifications are reversible, and the therapeutic effects of demethylating agents in hematological malignancies have been shown *in vitro* experiments and clinical trials [21]. Markers for monitoring the dose-response of such agents to minimize side-effects would be appreciated. The methylation status of the genes we present here could be used for these purposes and should be validated in larger studies.

In this study, we did not identify genes that were methylated in a lymphoma type or subtype specific pattern in patient samples, although the *BMP7* gene promoter was methylated in DLDCL ABC and not DLBCL GCB cell lines. This shows that the methylation pattern in cell lines not always reflects the methylation status in patient samples and underscores the need for validation. Interestingly, when we examined the methylation status of *DUSP4*, which was highly methylated across the various cell lines, we discovered that it was methylated in 50% of DLBCL ABC but was unmethylated in all DLBCL GCB. The possibility of *DUSP4* methylation as an additional biomarker for differentiating between the two DLBCL subtypes should be further investigated. Of interest, the *DUSP4* gene product dephosphorylates and thereby inactivates MAPKs, which are involved in regulation of growth and proliferation. Further, by blocking the MAPK cascade, *DUSP4* could act as a candidate tumor suppressor gene [26].

Taken together, we identified several genes (*BMPER, BMP7, CDH1, DUSP4* and *LRP12*) which were frequently methylated in major lymphoma types. In a future perspective, the hypermethylated *CDH1* and *LRP12* gene promoters could be used in a blood-based test to differentiate lymphoma patients from healthy donors and follicular hyperplasia.

## Supporting Information

File S1 **File includes Figures S1-S2 and Tables S1-S2.**
(PDF)
