# Supplementary material for: A Gene Panel, Including LRP12, Is Frequently Hypermethylated in Major Types of B-Cell Lymphoma
Source: PLoS One. 2014 Sep 16;9(9):e104249. doi: 10.1371/journal.pone.0104249 (PMC4165585; doi:10.1371/journal.pone.0104249)
Supplement: File S1 — File includes Figures S1-S2 and Tables S1-S2. (PDF) [file pone.0104249.s001.pdf]

Supplementary Text: Cell culture conditions

OciLy-3, -7 and -10 were cultured in IMDM medium (Invitrogen) supplemented with 20% human plasma (SeraCare Life Sciences, Inc. (California, USA)), 55 µM β-mercaptoethanol (Invitrogen), 100 Units/ml penicillin and 0.1 mg/ml streptomycin (PAA Laboratories) at 37°C with 5% CO_2. The remaining lymphoma cell lines were cultured in RPMI 1640 (PAA Laboratories, Austria), supplemented with 10% fetal calf serum (PAA Laboratories, Austria), 100 Units/ml penicillin and 0.1 mg/ml streptomycin (PAA Laboratories, Austria) at 37°C with 5% CO_2.

SF1:

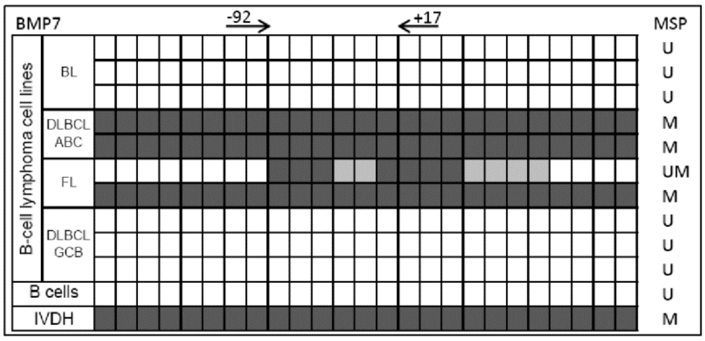

Supplementary Figure 1: Bisulfite sequencing of BMP7 gene promoter. CpG sites are represented by a box, a methylated site is symbolized by a dark grey box, a partially methylated CpG site is colored in gray and a white box represents an unmethylated CpG site. MSP-primer binding sites are indicated with an arrow above the CpG site. The distance from transcription start is indicated by the number above the arrow.

SF2:

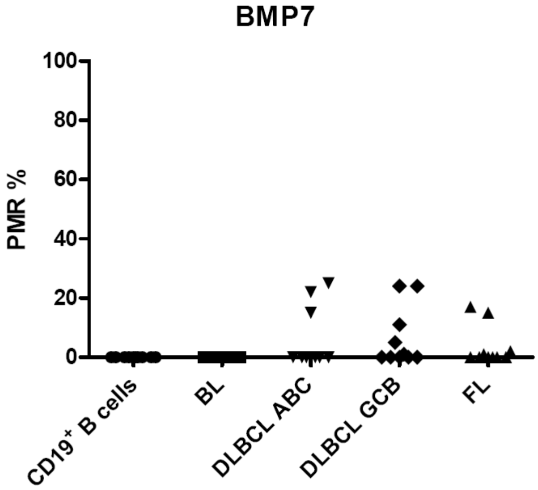

Supplementary Figure 2: Percent promoter methylation of *BMP7*. Each dot represents a healthy or lymphoma sample. Abbreviations: BL, Burkitt`s lymphoma; DLBCL ABC, activated B-cell like diffuse large B-cell lymphoma; DLBCL GCB, germinal centre B-cell like diffuse large B-cell lymphoma; FL, follicular lymphoma; PMR, percent methylated reference.

Supplementary Table 1: Primer and probe sequences for methylation analyses.

Oligo Name	Sequence (5'->3')	Length [mer]	TM
BCL2 mas	CCGAAAATCTCAAAAACTCG	20	53,2
BCL2 ms	TTTATGTTTGTGTTCGCGC	19	52,4
BCL2 uas	ACCAAAAATCTCAAAAACTCAAA	23	51,7
BCL2 us	AGATTTATGTTTGTGTTTGTGT	22	50,9
BCL2L10 mas	TACCGTAACCTAACGACCG	19	56,7
BCL2L10 ms	ATTATGGTCGATTCGTTGC	19	52,4
BCL2L10 uas	TCTACCATAACCTAACAACCAC	22	56,5
BCL2L10 us	ATTATTATGGTTGATTTGTTGT	22	49,1
BMP7 mas	TTATACGCCCTAAATCGCA	19	52,4
BMP7 ms	GAGGGGTTTTTCGAAGTTC	19	54,5
BMP7 uas	CCTTATACACCCTAAATCACA	21	54,0
BMP7 us	GGGAGGGGTTTTTTGAAGTTT	21	55,9
BMPER mas	TTCGAAAACTATCGCTATCG	20	53,2
BMPER ms	GGTGAGTTTACGTTTGTTGC	20	55,3
BMPER uas	TTCAAAAACTATCACTATCA	20	47,1
BMPER us	GGTGAGTTTATGTTTGTTGT	20	51,2
BSP BMP7 for	TTTAGAGGGAATGAATGAATTT	22	50,9
BSP BMP7 rev	CAACAAACCTAAAAATCCAAA	21	50,1
BSP BMPER for	GTTGGGGAATTGGAAGTT	18	51,4
BSP BMPER rev	TACRACAACCRAAAATAAACTAA	23	51,7
BSP CDH1 for	TTTGGGTGAAAGAGTGAGATT	21	54,0
BSP CDH1 rev	CAAACTCACAAATACTTTACAATTCC	26	56,9
BSP DUSP4 for	GGAGAATGATTATTTTTTGTTTAG	24	52,5
BSP DUSP4 rev	AATCTAAACTACCTACCRAACTC	23	56,2
BSP LRP12 for	GATTGGYGTTGTTTTGTT	18	48,0
BSP LRP12 rev	CRACCCTCCTAAAAACAA	18	50,3
CARL mas	ACCAATAATAATCGACCACG	20	47,7
CARL ms	GTGGAGATAGTTGCGTTTTC	20	49,7
CARL uas	ACCAATAATAATCAACCACA	20	43,6
CARL us	GTGGAGATAGTTGTGTTTTT	20	45,6
CCL22 mas	AATAAAAATATTTCACCACGCG	22	47,4
CCL22 ms	AATTGAGGTAGGTTTTTACGGC	22	51,1
CCL22 uas	CCAATAAAAATATTTCACCACACAC	25	51,1
CCL22 us	AGTAATTGAGGTAGGTTTTTATGGT	25	51,1
CDH1 mas	CCCCGTACCGCTAATTAACT	20	57,3
CDH1 ms	AGGGTTATCGCGTTTATGC	19	54,5
CDH1 uas	CCCCCCATACCACTAATTAACT	22	58,4
CDH1 us	TAGAGGGTTATTGTGTTTATGT	22	52,8
CLU_mas	ACCCTAAAATACGACCGC	18	53,7
CLU_ms	GGGATAGATAGTCGGGTTAATC	22	58,4
CLU_uas	AACCCTAAAATACAACCAC	19	50,2
CLU_us	TGGGATAGATAGTTGGGTTAATT	23	55,3
DUSP4 mas	GAAATACGACGACGAAAAAC	20	53,2
DUSP4 ms	AAAAATCGCGTGTTGTTC	18	49,1
DUSP4 uas	CACAAAATACAACAACAAAAAAC	23	51,7
DUSP4 us	TGGAAAAAATTGTGTGTTGTTT	21	50,1
GPSM2 mas	TAAACCAAAACTCGTAACGC	20	53,2
GPSM2 ms	AATGAGAGCGTTTGGAGTC	19	54,5
GPSM2 uas	CATAAACCAAAACTCATAACACC	23	55,3
GPSM2 us	TTGAATGAGAGTGTTTGGAGTT	22	54,7
HBEGF_mas	CGCAACTCGCTCTTCTTAA	19	54,5
HBEGF_ms	GGGTAGCGTTTATTCGGTC	19	56,7
HBEGF_uas	CACAACTCACTCTTCTTAA	19	50,2
HBEGF_us	GGGTAGTGTTTATTTGGTT	19	50,2
ICOSLG_mas	TACCCAACCGCATAATACGA	20	55,3
ICOSLG_ms	GCGGGAGCGTAGTTAGAGTC	20	61,4
ICOSLG_uas	CTACCCAACCACATAATACAA	21	54,0
ICOSLG_us	GGTGGGAGTGTAGTTAGAGTT	21	57,9
KLF13 mas	AATACCGAACGACAACGTTA	20	53,2
KLF13 ms	GTCGTTTATACGGAGTCGC	19	56,7
KLF13 uas	AATACCAAACAACAACATTAAA	22	49,1
KLF13 us	GGGTTGTTTATATGGAGTTGT	21	54,0
LRP12 mas	GTACGATCGACAATCCCCTA	20	57,3
LRP12 ms	ACGTTTTGTTATCGATTGGC	20	53,2

Oligo Name	Sequence (5'->3')	Length [mer]	TM
LRP12 uas	CCATACAATCAACAATCCCCTA	22	56,5
LRP12 us	GTATGTTTTGTTATTGATTGGT	22	50,9
MAP3K3 mas	TACCAATCATCGAAACAACG	20	47,7
MAP3K3 ms	AATGTGGGCGGAGTTTTAC	19	48,9
MAP3K3 uas	CTACCAATCATCAAAACAACACC	23	51,7
MAP3K3 us	GGGAATGTGGGTGGAGTTTTAT	22	53,0
MAP81P3 mas	CCCAACGCGCTATAACTTAA	20	61,0
MAP81P3 ms	GAACGGAGGTTTATTAGGGC	20	61,0
MAP81P3 uas	GGGAATGGAGGTTTATTAGGGT	22	56,0
MAP81P3 us	CCCCAACACACTATAACTTAAT	22	62,5
MYBL1_mas	AAAAACGAATTCCTACGCTC	20	53,2
MYBL1_ms	ACGTTTTTTTGGTGGGTC	18	51,4
MYBL1_uas	TAAAAAAACAAATTCCTACACTC	23	51,7
MYBL1_us	GAGATGTTTTTTTGGTGGGTT	21	54,0
NPY1R mas	ACAATCCTCGACTCCGAAA	19	54,5
NPY1R ms	TGGCGTTCGAGTTTTTTAGTC	21	55,9
NPY1R uas	CCCACAATCCTCAACTCCAAAA	22	58,4
NPY1R us	GGTTGGTGTTTGAGTTTTTTAGTT	24	55,9
PRKAR2B mas	AACGCCTACGACGCTAAC	18	56,0
PRKAR2B ms	TATTATACGGAGTAGACGCGC	21	57,9
PRKAR2B uas	CAAACACCTACAACACTAAC	20	53,2
PRKAR2B us	GTTATTATATGGAGTAGATGTGT	23	53,5
PTPRG mas	AACTTTAAAACCGACGAACG	20	53,2
PTPRG ms	GAGGGATTTAGCGTAAGGC	19	56,7
PTPRG uas	AACTTTAAAACCAACAAACA	20	47,1
PTPRG us	GAGGGATTTAGTGTAAGGT	19	52,4
SGPP2 mas	ACGAAAACAAAACCGCCTAT	20	53,2
SGPP2 ms	GGAGCGGTCGTAGGTGTAC	19	61,0
SGPP2 uas	ACCACAAAAACAAAACCACCTAT	23	55,3
SGPP2 us	ATGGGAGTGGTTGTAGGTGTAT	22	58,4
SNX22_mas	ACTAAAAACCCCGAAACG	18	51,4
SNX22_ms	TTGTGGGCGTATTAGATTTC	20	53,2
SNX22_uas	AACTAAAAACCCCAAAACA	19	48,0
SNX22_us	GTTGTGGGTGTATTAGATTTT	21	52,0
UCHL1 mas	TATAAAACGCCGACCAAAC	19	52,4
UCHL1 ms	TATTATTTCGCGTTGCGTAC	20	53,2
UCHL1 uas	CTATAAAACACCAACCAAAC	20	51,2
UCHL1 us	GTATTATTTTGTGTTGTGTAT	21	48,1
XRCC4 mas	CTTAACCGCCCGTCTTAATA	20	55,3
XRCC4 ms	TTTATTATAGCGAGGTCGGC	20	55,3
XRCC4 uas	CTTAACCACCCATCTTAATAAAC	23	55,3
XRCC4 us	TTTTTTATTATAGTGAGGTTGGT	23	51,7
qMSP BMP7 for	GAGGGGTTTTTCGAAGTTCGT	21	57,9
qMSP BMP7 Probe	GTTTAGGTTTTAGCGCGTATT	21	58,0
qMSP BMP7 rev	TCCCAACCTTATACGCCCTAAAT	23	58,9
qMSP BMPER for	AGAGTTTTCGTTGTAGTTATCGCGTAG	27	56,7
qMSP BMPER Probe	CGAGCGCGTTTCG	13	58,0
qMSP BMPER rev	TCGAAATAGTAGCGGTAGTTCGG	23	55,3
qMSP CDH1 for	AATTTTAGGTTAGAGGGTTATCGCGT	26	60,1
qMSP CDH1 Probe	CGCCCACCCGACCTCGAT	18	71,0
qMSP CDH1 rev	TCCCCAAAACGAAACTAACGAC	22	58,4
qMSP DUSP4 for	TGGAGGGATTTGGCGTTC	18	50,3
qMSP DUSP4 Probe	TTCGCGGTTTTCGGGT	16	62,0
qMSP DUSP4 rev	CGCGCGGGTAGGGTTT	16	51,1
qMSP LRP12 for	GTTTTGTTATCGATTGGCGTTG	22	51,1
qMSP LRP12 Probe	TGGGTTTTCGTCGCGTGG	18	67,0
qMSP LRP12 rev	GTACGATCGACAATCCCCTAACC	23	57,1

Supplementary table 2: Statistical evaluation of potential differences between the groups analyzed by qMSP in figure 2

	**CDH1**	**LRP12**	**BMPER**	**DUSP4**
CD19+ B cells vs FH	0.0883	0.0107	0.404	0.939
CD19+ B cells vs PBMC	< 0.0001	0.0001	0.1134	1.0000
CD19+ B cells vs Tonsils	0.6749	0.0055	0.4152	0.9421
CD19+ B cells vs DLBCL ABC	<0.0001	<0.0001	0.0008	0.0491
CD19+ B cells vs DLBCL GCB	<0.0001	<0.0001	0.0003	0.2327
CD19+ B cells vs FL	<0.0001	<0.0001	0.0065	n.a.
CD19+ B cells vs PMBL	0.0003	0.0003	0.0278	0.0640
FH vs PBMC	0.0421	0.1187	0.4406	1.0000
FH vs Tonsils	0.4124	0.8282	1,0000	0.939
FH vs DLBCL ABC	0.0001	< 0.0001	0.0043	0.0642
FH vs DLBCL GCB	<0.0001	0.0001	0.0017	0.2735
FH vs FL	0.0001	0.0001	0.0156	n.a.
FH vs PMBL	0.0017	0.0016	0.0393	0.082
PBMC vs Tonsils	0.0079	0.1687	0.5036	1.0000
PBMC vs DLBCL ABC	< 0.0001	< 0.0001	0.0015	0.0491
PBMC vs DLBCL GCB	< 0.0001	< 0.0001	0.0005	0.2327
PBMC vs FL	< 0.0001	< 0.0001	0.0055	n.a.
PBMC vs PMBL	0.0013	0.0006	0.0147	0.064
Tonsils vs DLBCL ABC	< 0.0001	< 0.0001	0.0031	0.0491
Tonsils vs DLBCL GCB	< 0.0001	< 0.0001	0.0012	0.2327
Tonsils vs FL	< 0.0001	< 0.0001	0.0106	n.a.
Tonsils vs PMBL	0.0013	0.0012	0.0292	0.064
DLBCL ABC vs DLBCL GCB	0.2339	0.9862	0.3056	0.2836
DLBCL ABC vs FL	0.9831	0.4212	0.1123	n.a.
DLBCL ABC vs PMBL	0.8676	0.1823	0.6609	0.5350
DLBCL GCB vs FL	0.0993	0.2962	0.0263	n.a.
DLBCL GCB vs PMBL	0.3196	0.2532	0.2212	0.3709
FL vs PMBL	0.9626	0.3736	0.4743	n.a.
CD19+ B cells test vs validation	0.084	0.615	0.435	n.a.
DLBCL ABC test vs validation	0.83	0.586	0.225	n.a.
DLBCL GCB test vs validation	0.417	0.82	0.554	n.a.
FL test vs validation	0.707	0.843	0.755	n.a.

P values are derived from a 2-sided Mann-Whitney test
